# Efficient carbon and nitrogen transfer from marine diatom aggregates to colonizing bacterial groups

**DOI:** 10.1038/s41598-022-18915-0

**Published:** 2022-09-02

**Authors:** Nestor Arandia-Gorostidi, Hugo Berthelot, Federica Calabrese, Hryhoriy Stryhanyuk, Isabell Klawonn, Morten Iversen, Nurun Nahar, Hans-Peter Grossart, Helle Ploug, Niculina Musat

**Affiliations:** 1 Department of Isotope Biogeochemistry, Helmholtz-Centre for Environmental Research (UFZ), Permoserstrasse 15, 04318 Leipzig, Germany; 2 Department of Earth System Science, Stanford University, Green Earth Sciences Building, 367 Panama St., Room 129, Stanford, CA 94305-4216 USA; 3 Department of Marine Sciences, University of Gothenburg, Gothenburg, Sweden; 4 Laboratoire des Sciences de l’Environnement Marin (LEMAR), UMR 6539 UBO/CNRS/IRD/IFREMER, Institut Universitaire Européen de la Mer (IUEM), Brest, France; 5 IFREMER, DYNECO, Pelagos Laboratory, Plouzané, France; 6 Department of Organismic and Evolutionary BiologyBiological Laboratories, Harvard University, 16 Divinity Avenue, Cambridge, MA USA; 7 Department of Ecology, Environment and Plant Sciences, Stockholm University, 10691 Stockholm, Sweden; 8 Leibniz Institute for Baltic Sea Research (IOW), Rostock, Germany; 9 Alfred Wegener Institute, Helmholtz Center for Polar and Marine Research, Bremerhaven, Germany; 10 Marum and University of Bremen, Bremen, Germany; 11 Department of Plant Biology and Forest Genetics, Swedish University of Agricultural Sciences, Uppsala, Sweden; 12 Biological and Environmental Sciences, University of Gothenburg, Box 461, 40530 Gothenburg, Sweden; 13 Institute for Biochemistry and Biology, Potsdam University, Potsdam, Germany; 14 Department Plankton and Microbial Ecology, Leibniz Institute for Freshwater Ecology and Inland Fisheries, Berlin/Stechlin, Germany

**Keywords:** Microbial biooceanography, Element cycles, Water microbiology

## Abstract

Bacterial degradation of sinking diatom aggregates is key for the availability of organic matter in the deep-ocean. Yet, little is known about the impact of aggregate colonization by different bacterial taxa on organic carbon and nutrient cycling within aggregates. Here, we tracked the carbon (C) and nitrogen (N) transfer from the diatom *Leptocylindrus danicus* to different environmental bacterial groups using a combination of ^13^C and ^15^N isotope incubation (incubated for 72 h), CARD-FISH and nanoSIMS single-cell analysis. *Pseudoalteromonas* bacterial group was the first colonizing diatom-aggregates, succeeded by the *Alteromonas* group. Within aggregates, diatom-attached bacteria were considerably more enriched in ^13^C and ^15^N than non-attached bacteria. Isotopic mass balance budget indicates that both groups showed comparable levels of diatom C in their biomass, accounting for 19 ± 7% and 15 ± 11%, respectively. In contrast to C, bacteria of the *Alteromonas* groups showed significantly higher levels of N derived from diatoms (77 ± 28%) than *Pseudoalteromonas* (47 ± 17%), suggesting a competitive advantage for *Alteromonas* in the N-limiting environments of the deep-sea. Our results imply that bacterial succession within diatom aggregates may largely impact taxa-specific C and N uptake, which may have important consequences for the quantity and quality of organic matter exported to the deep ocean.

## Introduction

Macroscopic aggregates, known as marine snow, play a key function in the transport of organic matter from the upper layer of the oceans to the deep sea^1^. These sinking aggregates are mostly formed during diatom blooms and their decay^2,3^ as a consequence of the production of sticky transparent exopolymer particles^4,5^. Diatom aggregates are rapidly colonized by a complex and highly dynamic pool of microorganisms^6,7^ which strongly impact the formation and degradation of sinking diatom aggregates^8,9^. Among these microorganisms, heterotrophic bacteria are major players controlling the particle breakdown through exoenzymatic activity^1,10–12^, mediating the transfer of particulate matter to the dissolved pool in the mesopelagic zone^13,14^.

The ability of microbes to detect and exploit sinking diatom aggregates largely determines the efficiency of particle colonization^15–17^. Generally, heterotrophic bacterial taxa colonizing the aggregates are more diverse than free-living cells^18–21^ and have higher metabolic activities than their free-living counterparts^6,10,22^. In particular, *Flavobacteria* and some *Gammaproteobacteria* (including *Alteromonas* and *Pseudoalteromonas* groups) due to their chemotactic and attachment abilities^23,24^ are prominent bacterial groups found in association with marine particles^15,24–28^, with an important contribution to aggregate degradation in the dark ocean^29^. In recent studies^20,30^, it has been shown that colonization of sinking particles may act as a vector inoculating viable cells into the meso- and bathypelagic zones, with a potentially large impact on the microbial community composition of the deep sea^30^. Yet, our understanding of the importance and role of different bacterial groups in the C and N cycling within the aggregates and, hence, determining the amount of C and N exported to the surrounding water is still limited.

We combined stable isotope probing with taxonomic identification techniques aiming to analyze the dynamics in diatom aggregate colonization by different phylogenetic groups of bacteria, as well as their use of diatom-derived C (DDC) and N (DDN). We performed roller tank incubation experiments (72 h) by inoculating a ^13^C and ^15^N pre-labelled aggregate-forming diatom culture (*Leptocylindrus danicus*) with natural seawater samples containing diverse bacterial communities. We followed bacterial aggregate colonization and use of DDC and DDN using single-cell analyses based on Catalyzed Reported Deposition-Fluorescence In Situ Hybridization (CARD-FISH) and nano-scale Secondary-Ion Mass Spectrometry (nanoSIMS). Our data show that diatom colonization was mostly dominated by two *Gammaproteobacteria* groups, *Pseudoalteromonas* and *Alteromonas*, which followed a distinct succession and DDC/DDN incorporation pattern, suggesting a specific role of each taxonomic group in the colonization and degradation of marine aggregates.

## Results

### Dynamics of bacteria within the aggregates

**Figure 1:**
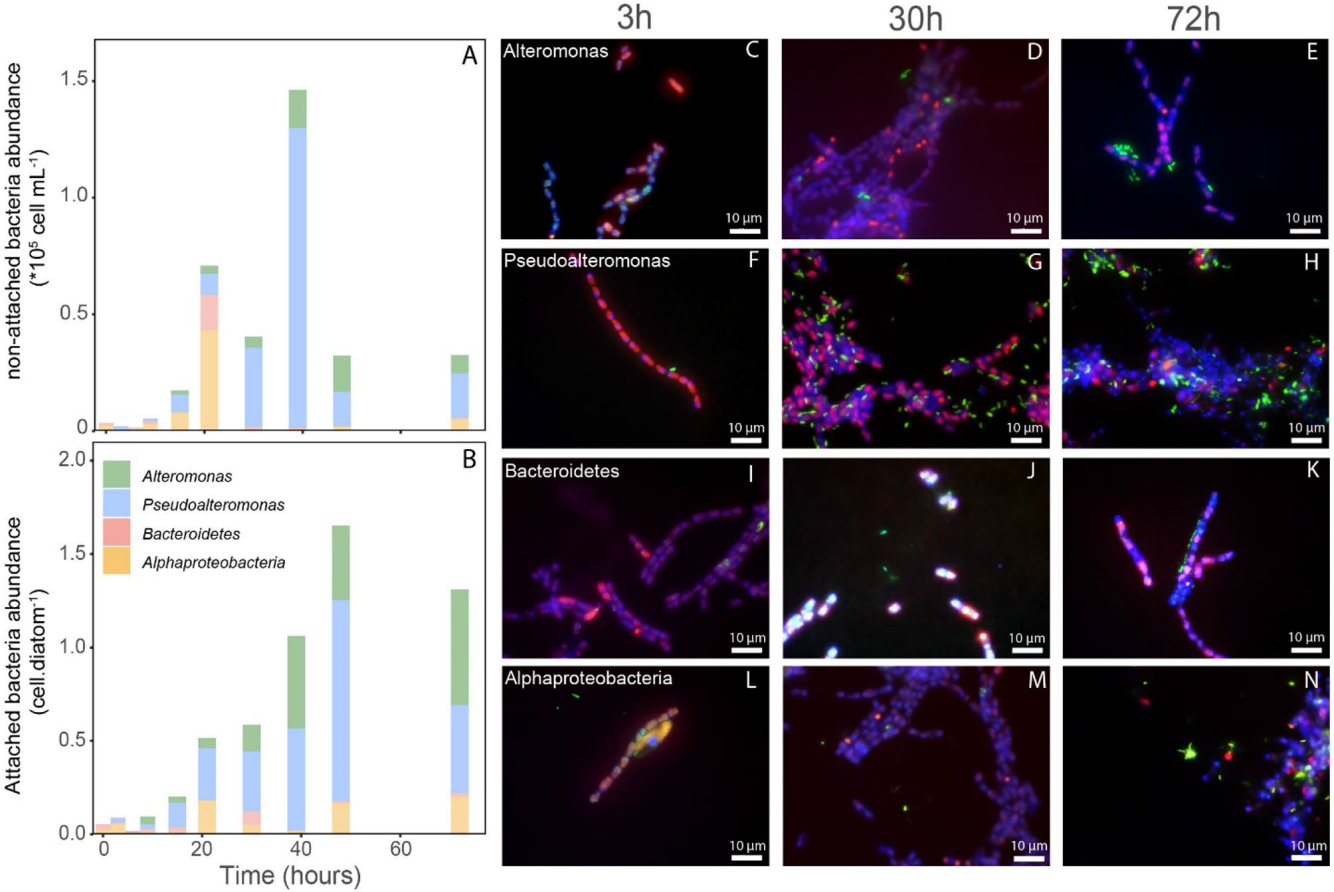
Bacterial abundances of various groups within aggregates targeted by CARD-FISH as a function of incubation time (h) for the non-attached (**A**) and attached fractions (**B**, expressed as number of bacterial cells per diatom). Images in the right panel show epifluorescence microscopy images at different time points for *Alphaproteobacteria* (**C**–**E**), *Alteromonas* (**F**–**H**), *Bacteroidetes* (**I**–**K**) and *Pseudoalteromonas* (**L**–**N**) (depicted in green after CARD-FISH with Alexa488-labelled tyramides) (non-)attached to diatom filaments (depicted in red due to their natural chlorophyll autofluorescence). Blue color is given by counterstaining with DAPI of hybridized samples.

A ^13^C and ^15^N pre-labelled *L. danicus* culture was inoculated with an environmental non-labelled microbial community collected at 50 m water depth in the Atlantic Ocean (Cape Blanc, Mauretania). Aggregation experiment was done in roller tanks. In this work, we differentiate between bacteria that were physically attached to the diatom cells from those that were found within the aggregates (and hence were not free-living), but not directly attached to diatoms (here on called non-attached). An example of the visual identification of both bacterial fractions is shown in Supplementary Fig. S1. Both, attached and non-attached fractions were detected by CARD-FISH hybridization and counted manually by epifluorescence microscopy analysis. At the start of the experiment, we detected only a few bacteria (< 0.1 bacteria diatom^−1^) associated to the initially small diatom aggregates (< 0.5 mm), among which 23% of the bacteria were directly attached to the diatoms (Fig. 1). Macroaggregates (> 1 mm) were formed a few hours (< 3 h) after the initiation of the experiment. During the experiment, bacterial abundances within the formed aggregates increased, reaching to > 1 bacterium diatom^−1^ after 30 h and stabilized around this value until the end of the experiment (72 h). Due to the heterogeneous nature of diatom aggregates, the bacterial abundances were highly variable (coefficient of variation of 84% on average, ranging between 21 and 143%).

**Figure 2:**
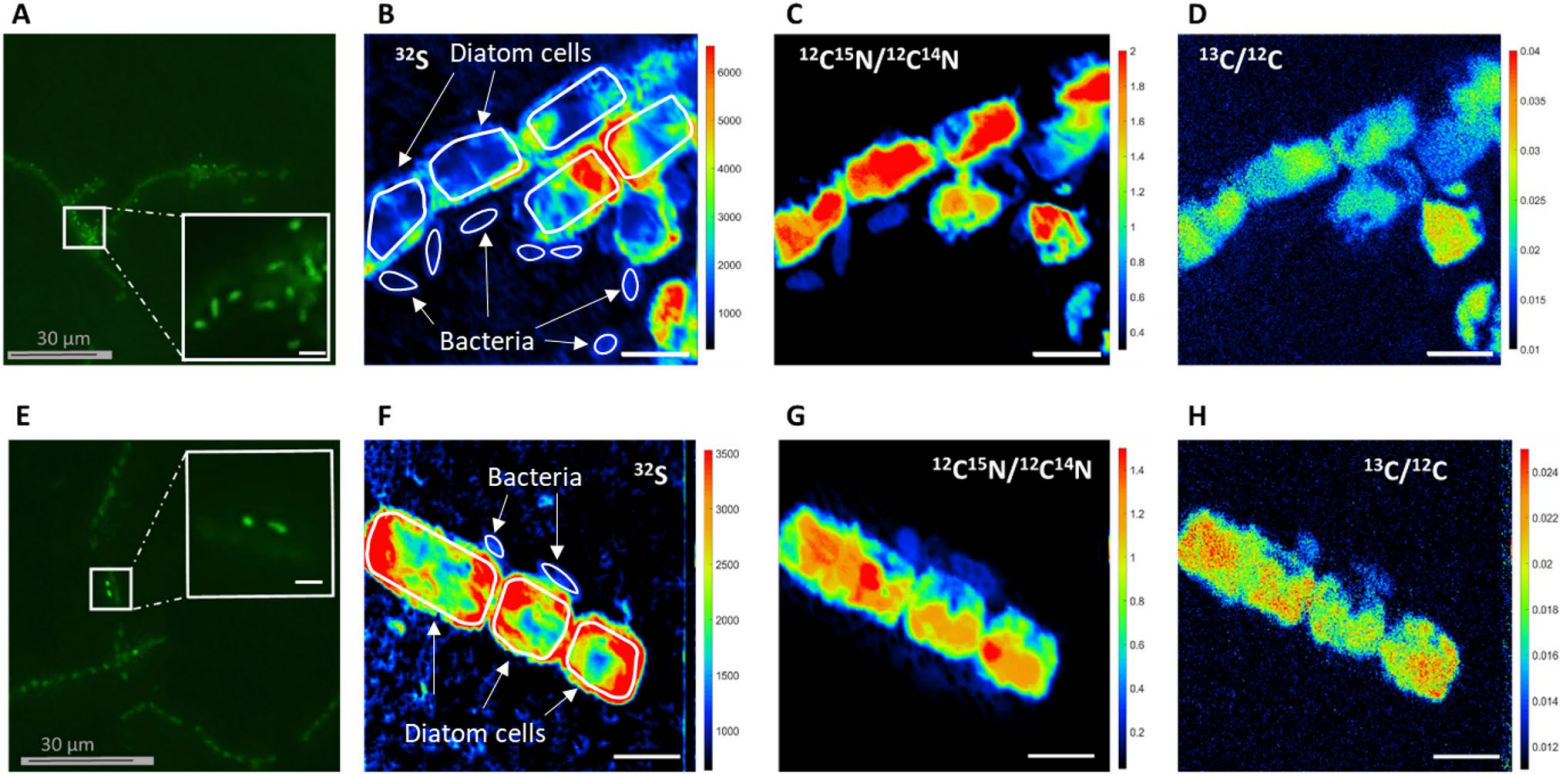
Correlative CARD-FISH and nanoSIMS imaging of *Alteromonas* (**A**–**D**) and *Pseudoalteromonas* spp. (**E**–**H**), sampled at 72 h. (**A**, **E**) Fluorescence micrographs acquired after CARD-FISH but prior to the nanoSIMS analysis; the contoured insets represent a zoom-in of the fields of view (FoV) that are shown in the nanoSIMS images (**B**–**H**). **B**–**C**–**D** and **F**–**G**–**H** represent the chemical maps acquired during the nanoSIMS analysis for Sulfur (^32^S), as an indicator of biomass, ^12^C^15^N/^12^C^14^N and ^13^C/^12^C for quantifying the nitrogen and carbon enrichment, respectively. Contours of diatom cells and bacteria are drawn in panels B and F only. Scale bars in the insets and chemical maps are 3 µm.

At the start of the experiment, only *Alphaproteobacteria* and *Bacteroidetes* were associated with *L. danicus* within the aggregates, although at low abundances (< 0.05 cells diatoms^−1^, Fig. 1, Table S1). The abundances of these groups stayed below 0.2 cells diatom^−1^ during the entire experiment (72 h, Fig. 1). The *Gammaproteobacteria Alteromonas* and *Pseudoalteromonas* were not detected at the beginning of the experiment but showed a fast accumulation within the aggregates over time, reaching up to 0.6 and 1.1 cells diatom^−1^, respectively. As a result, the bacterial community shifted from *Bacteroidetes* and *Alphaproteobacteria* dominance toward a *Gammaproteobacteria* dominance. Overall, the proportion of cells attached vs. non-attached diatoms (bacteria that are within the aggregates but with no physical contact with the diatom cells) to diatoms was significantly higher (p < 0.01, Wilcoxon test) for *Pseudoalteromonas* (51%) and *Alteromonas* (32%) than for *Alphaproteobacteria* (14%) and *Bacteroidetes* (4%) (Fig. 2).

### Phytoplankton and bacterial cell-specific isotope enrichment

**Figure 3:**
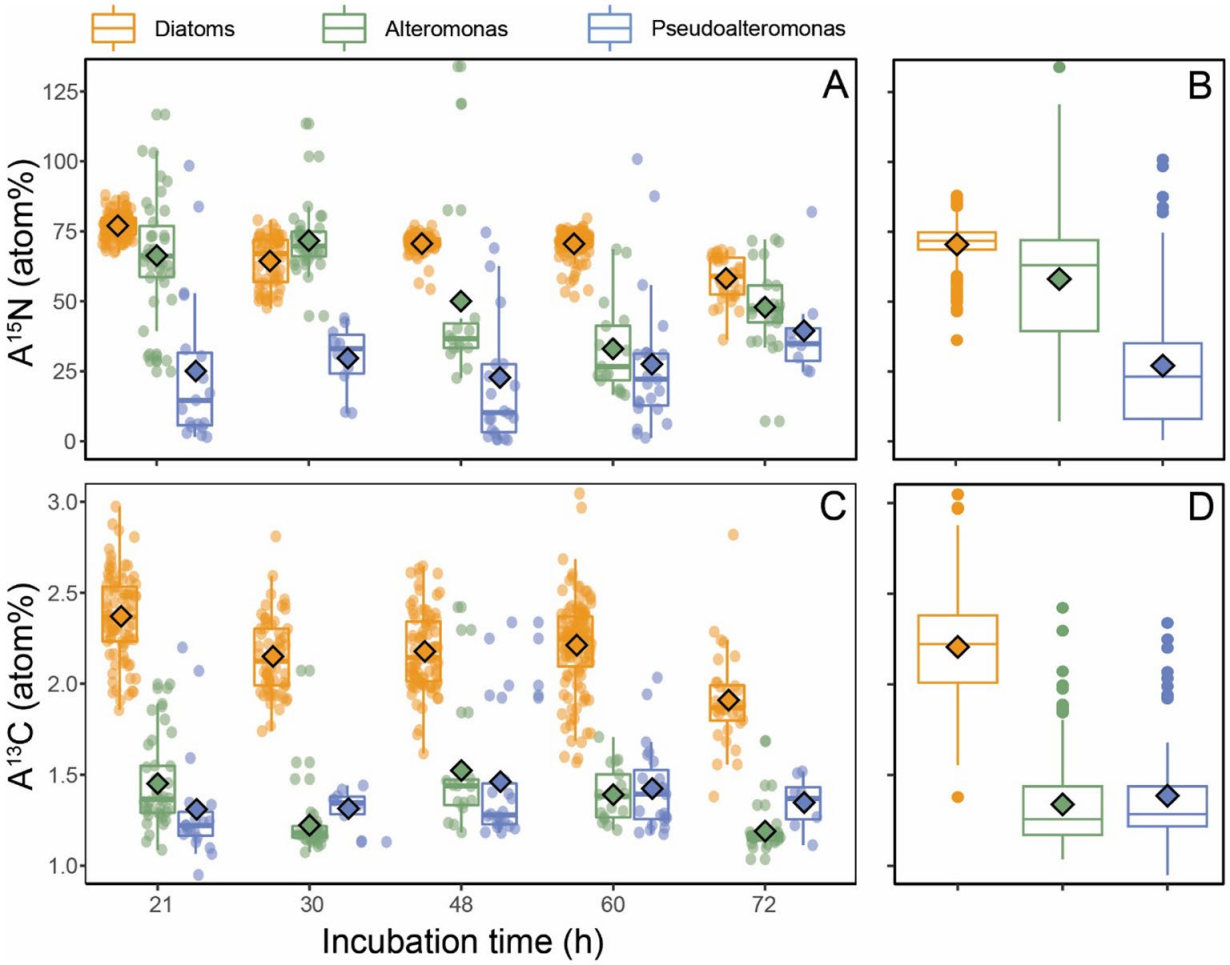
(**A**, **C**) ^15^N and ^13^C isotopic enrichment (atom%) of the diatom *L. danicus* and bacteria *Alteromonas* and *Pseudoalteromonas* combining both, attached and non-attached fractions, as a function of time. Each circular dot represents an analyzed cell (total cells analyzed 621: 131 Alteromonas, 82 Pseudoalteromonas and 407 diatoms) and larger dots represent groups average at each time point. (**B**, **D**) Whisker plots of the ^15^N and ^13^C isotopic enrichments for each group analyzed (as average for all times points included).

To analyze the transfer of ^13^C and ^15^N from pre-labelled diatom aggregates to bacteria, a total of 407 diatom cells and 213 bacteria were quantified at single-cell level using nanoSIMS. For the nanoSIMS analysis, we only focused on *Alteromonas* and *Pseudoalteromonas* groups, as both groups represented most of the aggregate-associated bacterial cells as observed in Fig. 1. The high number of cells analyzed allowed us to calculate the proportion of diatom-derived carbon (DDC) and nitrogen (DDN) in individual bacterial cells. At the beginning of the experiment, diatom fractional isotope abundances for C and N (A^13^C and A^15^N, respectively) averaged 2.3 ± 0.1 and 66.4 ± 6.2 atom% (Fig. 3A,C) but decreased linearly to 1.9 ± 0.41 and 54.4 ± 21.6 atom%, respectively, after 72 h. Considering the low natural fractional isotopic abundance of ^13^C (1.08 atom%) and ^15^N (0.37 atom%), this corresponded to a decrease of 35% and 18% of their isotopic enrichment, respectively. Significant ^13^C and ^15^N isotopic enrichments were also detected in bacterial cells. While *Alteromonas* showed higher A^15^N during the first time points (21 h and 30 h) compared to the last time points (48, 60, and 72 h, Fig. 3), the maximum A^15^N for *Pseudoalteromonas* was detected at 72 h and was significantly higher than A^15^N at 21 h and 30 h (Wilcoxon test, p-value = 0.03). When averaged over time, A^13^C was not significantly different between *Alteromonas* (1.19 ± 0.16 atom%) and *Pseudoalteromonas* (1.21 ± 0.16 atom%) (Wilcoxon test, p = 0.47), while A^15^N was significantly higher for *Alteromonas* (25.65 ± 7.21 atom%) than for *Pseudoalteromonas* (14.05 ± 3.01 atom%) (Wilcoxon test, p < 0.001). The difference in bacterial A^15^N between both bacterial groups was highest at the beginning of the incubation (twofold) and tended to be reduced towards the end of the incubation (1.2-fold, Fig. 3). Bacterial cells of both groups were on average more enriched in ^13^C and ^15^N when physically attached to the diatoms rather than solely embedded in the aggregate (Wilcoxon test, p < 0.001) (Table 1). This difference was most noticeable for *Alteromonas*: at all time points, attached *Alteromonas* cells were significantly more enriched in ^13^C and ^15^N compared to non-attached ones while for *Pseudoalteromonas* the difference was only significant after 72 h for both ^13^C and ^15^N (Fig. 3).

**Table 1:** Isotopic abundances presented as fractions of identified bacteria (average ± standard deviation) including all time points.

	A^13^C	A^15^N	Cell numbers
	(Average ± standard deviation)	(Average ± standard deviation)	
***Alteromonas***			
Attached	1.36 ± 0.20	66.98 ± 9.00	50
Non-attached	1.13 ± 0.04	44.80 ± 4.36	81
***Pseudoalteromonas***			
Attached	1.39 ± 0.12	41.66 ± 10.98	13
Non-attached	1.25 ± 0.14	27.60 ± 8.80	69

Attached bacteria are significantly more isotopically enriched than non-attached ones for both phylogroups and both isotopes (Wilcoxon–Mann–Whitney, p < 0.05).

**Figure 4:**
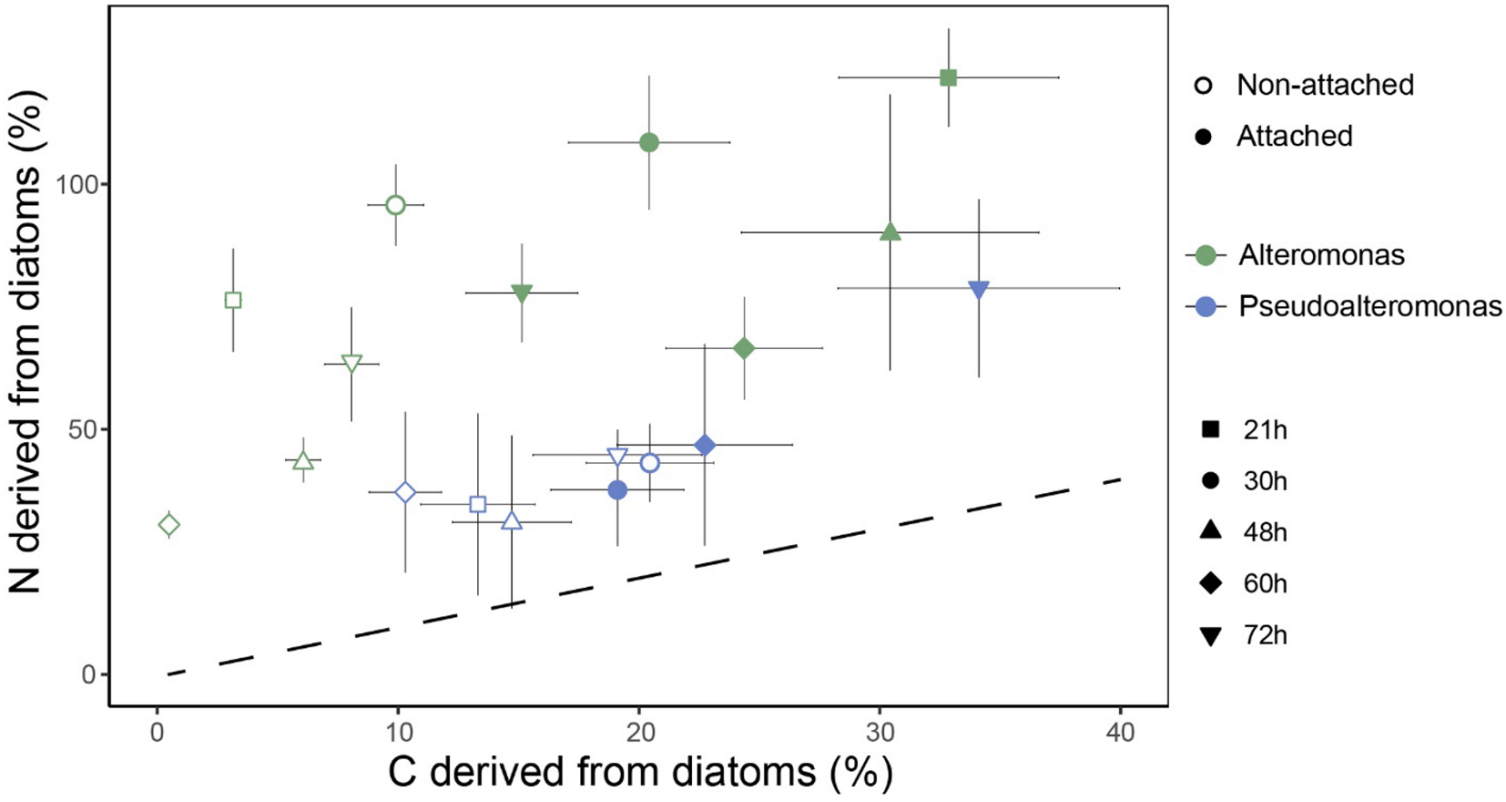
Averaged proportion of diatom-derived N (DDN) as a function of diatom-derived C (DDC) in the biomass of *Pseudoalteromonas* (blue dots) and *Alteromonas* (green dots) cells in aggregates physically attached (filled dots) or non-attached (empty dots) to diatoms (213 bacteria analyzed in total). Each dot represents one of the five time points. Dashed line indicates a 1:1 ratio between DDC/DDN.

Using a mass balance approach, we calculated that DDC and DDN represented on average 17% (1–34%) and 63% (31–122%) of the aggregate-embedded bacterial C and N biomass, respectively (Fig. 4). The DDC was similar for both strains, whereas DDN was higher for *Alteromonas* than for *Pseudoalteromonas* (Fig. 4, Table S2). The low DDC:DDN ratio for both *Pseudoalteromonas* and *Alteromonas* indicated a preferential diatom-derived N uptake for both groups, particularly of *Alteromonas*, which showed an average DDC:DDN ratio consistently lower than *Pseudoalteromonas* (0.18 and 0.43, respectively).

## Discussion

Heterotrophic bacteria living in association with sinking marine aggregates hydrolyze particulate organic matter (POM) to dissolved organic matter (DOM), releasing it to the surrounding water. Combined, bacterial hydrolyzation and respiration of organic matter cause the attenuation of the export of organic matter to deeper depths, lowering the efficiency of the biological carbon pump^13^. Previous studies have shown that attached heterotrophic bacteria drive C and N turnover^31,32^ and the breakdown of aggregates through enzymatic hydrolysis^33,34^. Yet, due to the small spatial scale in which these processes occur, bulk analysis cannot accurately determine the importance of these diatom-bacteria associations on C and N cycling within aggregates vs. those in the surrounding water (so-called free-living bacteria). In this work, we have coupled nanoSIMS and CARD-FISH single-cell analysis, to assess the role of phylogenetically different bacteria in the colonization of diatom cells and in the C and N cycling within diatom aggregates. Here, we differentiate between attached cells (the ones that are in physical contact with the diatoms) from the non-attached ones (bacteria within the aggregates but with no physical contact with the diatom cells). In contrast to free-living bacteria, the non-attached cells are found within the aggregates, most likely embedded within the organic matrix of the aggregates such as transparent exopolymer particles (see Figs. S1, S3). Using a ^15^N and ^13^C pre-labelled axenic *L. danicus* culture, which is described as an important aggregate-forming diatom^4^, and fresh field-collected bacteria, we show the importance of a fast aggregate colonization ability and the following bacterial succession for an efficient C and N assimilation within the aggregates.

The increasing bacterial abundances on diatom aggregates observed during our roller tank incubations are in good agreement with previous reports^35–37^. Due to the rapid aggregate colonization by non-attached bacteria in the first hours of incubation^36^ and the following high growth-efficiency of aggregate-associated bacteria observed at the early colonization phase^38^, it is likely that the increasing bacterial abundances resulted from a combination of colonization and active growth within the aggregates. For example, high attachment and subsequent colonization rates would explain the switch of *Pseudoalteromonas* abundance from the non-attached to the attached fraction between 39 and 48 h of incubation. On the other hand, the consistently higher abundance of *Alteromonas* in the attached fraction compared to non-attached and their high N uptake may suggest an active growth within the aggregates. Yet, aggregate colonization by the low abundant non-attached *Alteromonas* was also likely happening. Regardless of the process responsible for the increase in colonizer abundance of the attached fraction, we derived that the net bacterial abundance increase would be 6 d^−1^ during the first 30 h of incubation. This is one order of magnitude higher than most of the estimates for marine free-living bacteria (~ 0.2–0.4 d^−1^^39,40^) but closer to the range of reported values for aggregate-attached bacteria (0.5 to > 4 d^−1^^7^).

Bacterial colonization is mediated by the ability of bacteria to detect and exploit marine aggregates^41^. Both *Alteromonas* and *Pseudoalteromonas* dramatically increased in abundance within the diatom aggregates and are well known to colonize marine particles by chemotactic motility^24,42,43^. They have also been shown to efficiently use algal-derived organic matter^44,45^. Yet, such abilities have been more frequently described in other bacterial groups such as *Flavobacteria* or *Roseobacter*^15,46,47^, while in the current work, these groups were less frequently attached to the diatom aggregates than members of the *Pseudoalteromonas* and *Alteromonas* groups. The observed high variability in the composition of the aggregate-associated bacterial community may be related to the distinct microbial taxa harbored by different phytoplankton species^45,48^. Another possibility is that both groups have better adaptations to efficiently colonize diatom-aggregates, in particularly *Pseudoalteromonas*, whose physiological abilities to live attached to aggregates would provide a competitive advantage against other members of the aggregate-associated community^42^. The subsequent changes in attached community composition, with increasing *Alteromonas* abundance in the attached fraction after 39 h, could be related to myriad microbial interactions that happen in the aggregates, including viral lysis or bacterial competitive interactions^55^, that may control colonization succession.

Bacteria growing within aggregates show a higher exoenzymatic activity compared to free-living bacteria, which facilitates the access to more recalcitrant DOM^1,49^. The fast colonization ability of *Pseudoalteromonas* also suggests that they may hold a key role during the initial phase of aggregate hydrolysis by exoenzymatic activity, facilitating the subsequent aggregate colonization by other microbial taxa. The micro-scale succession of colonizing taxa within marine aggregates has been previously described^35^, however, our study suggests that *Pseudoalteromonas* may be one of the first responsible for the diatom-aggregate breakdown, which may impact the C and N assimilated by other taxa.

Monitoring the isotopic composition of bacteria at different timepoints allowed us to further investigate the relative importance of colonization and in situ microbial growth. In stable isotope probing experiments, the isotopic ratio of the targeted organisms is expected to increase with time, until an isotopic equilibrium is reached (i.e., when the isotope ratio of targeted organisms equals that of the isotope source pool, see model in Fig. S2). We observed a considerable variability in the isotopic enrichment over time, with *Alteromonas* A^15^N as high as the diatoms A^15^N after 21 h but decreasing thereafter. The isotopic equilibrium reached within the first hours of the incubation demonstrated the efficient use of DDN by *Alteromonas*. The counterintuitive decrease in DDN between 30 and 72 h of the incubation could result from fast microbial turnover rates (with rates of bacterial colonization and detachment as fast as 3 h^50^), which would prevent the observation of a continuous increase of their isotopic composition. However, it is also possible that besides the diatom derived ^15^N, bacteria used alternative non-labelled sources of N. A recent study has shown that marine aggregates may constitute oxygen depleted microenvironments favorable for microbial N_2_ fixation^51^. According to the authors, such microhabitats would need around 2 days to develop, which falls in good agreement with the decrease in A15N observed for *Alteromonas* and indicates that the *Alteromonas* group may have used the recently fixed and non-labelled N in the latest phase of the incubation. Despite the observed trends, our results suggest that *Alteromonas* group is able to take up a large fraction of DDN within a few hours of aggregate formation. Such fast N uptake ability may have an important impact on the N circulation and remineralization within the first stages of aggregate sinking, retaining a big fraction of the aggregate derived N in the surface ocean.

While *Alteromonas* represented most of the attached bacteria cells detected after 15 h of incubation, the relatively low DDN of *Pseudoalteromonas* (38% after 30 h) implies that alternative non-labelled N sources play a substantial role for the build-up of bacterial biomass. Although parts of this N uptake could derive from N-fixation, the time needed for the formation of an anoxic microenvironment within the aggregates^51^ does not explain the initial low DDN. Thus, the relatively low DDN in *Pseudoalteromonas* may have been related to other sources rather than those derived from N-fixation, such as dissolved N incorporated from the seawater. We cannot disregard the impact of grazers and viral infections on the incorporation of non-labelled substrates, as bacterial lysis through grazing or virus activity could also recirculate dissolved organic substrates^52–54^. Despite the origin of both, non-labelled and ^15^N-labelled substrates, our results clearly show distinct colonization strategies by *Pseudoalteromonas* and *Alteromonas*. *Pseudoalteromonas* may have incorporated a high amount of non-labelled N at the beginning of the incubation in order to gather enough energy for a rapid aggregate colonization. On the other hand, *Alteromonas* was more dependent on the aggregate-derived ^15^N, delaying the aggregate colonization to a secondary phase.

In contrast to DDN, we found that cell-specific DDC incorporation rates were very similar for both analyzed bacterial groups. This consistent C uptake agrees with a previous study showing similar algal-derived dissolved organic C (DOC) uptake by two copiotrophic groups associated with the diatom phycosphere^47^, suggesting that C assimilation heterogeneity in aggregate-attached bacteria is relatively low and barely varies between different phylogenetic groups. Another interesting observation is that the cells of the attached fraction were able to incorporate substantially more C than the non-attached ones, while no difference was observed for N (Fig. 4). This result is in good agreement with previous observations where higher isotope uptake was observed in bacteria physically attached to phytoplankton cells than in their free-living counterparts^55^. Despite we did not target the free-living fraction in this study, current results further highlight that physical contact between bacteria and phytoplankton is the most effective way of nutrient transfer. Similar observation regarding close physical contact as a requirement for efficient nutrient transfer was shown for other types of interactions e.g. fungi-bacteria, where a proximity of 1.7 µm was necessary for efficient nutrient and water transfer from fungi to bacteria while bacterial cells further away could not receive the nutrients provided by the fungi mycelium, remaining metabolically inactive^56^.

The low C assimilation by both bacterial groups studied here was also remarkable, representing only 10–30% of DDC. According to our calculations (and considering an average of 1 bacteria per diatom), at this rate, total particle degradation would take 10 times longer than previous estimations (8–9 days^31^). Such low C assimilation in comparison to N may be a consequence of different metabolic pathways for distinct compounds. While the majority of the incorporated N was assimilated as biomass, it is possible that a large fraction of DDC was metabolized and remineralized as CO_2_ through bacterial respiration. In fact, high respiration rates have been measured in aggregates (with C turnover rates of 0.1–0.2 d^−1^^57^) releasing most of the DDC back to the system as CO_2_; microbial grazing and viral lysis would explain the low microbial abundance despite the potentially high respiration rates^32,54^. Another possibility for the difference observed between DDN and DDC is a preferential decomposition of particulate organic nitrogen (PON) as compared to carbon, as previously suggested^34,58^, which falls in good agreement with increasing C:N ratio with increasing depth, and is generally observed in sinking materials across the Oceans^59^. According to our results, N would be degraded twice as fast as C within the aggregates, meaning these latter would become more C-rich while sinking (also supported by previous observations^33^). Thus, contrary to the initial hypothesis, bacterial degradation would change the stoichiometry of the sinking particles, marginally increasing the efficiency of the biological carbon pump.

The current work highlights the importance of a fast colonization ability for an efficient C and N assimilation by bacteria within diatom aggregates. The fast bacterial turnover, together with the high uptake of diatom-derived substrates (mostly N) within the first 21 h of incubation, strongly indicate that the first colonization phase is a key step in C and N cycling between diatoms and associated heterotrophic bacteria. The imbalance that we observed between the use of DDN and DDC further highlights the importance of microbial succession within the aggregates on the C export to the deep ocean. However, how other factors, such as increasing pressure^60,61^ and decreasing temperature^62^ with increasing depth, may impact the C and N assimilation by aggregate-associated bacteria should be further addressed to understand the element cycling in the meso and bathypelagic realm. Nevertheless, our results represent an important step forward in determining the impact of cell-specific colonization on the N and C cycling within settling diatom aggregates.

## Material and methods

### Diatom cultures and incubation with stable isotopes.

*Leptocylindrus danicus* (strain CCMP470) were cultured in a f/2 medium and grown at constant temperature^63^. The medium was prepared with ^13^C-bicarbonate (NaH^13^CO_3_, Sigma Aldrich) and ^15^N-nitrate (^15^NO_3_^−^, Sigma Aldrich), in order to isotopically label the diatom biomass during growth (time of growth with the label). The final ^13^C and ^15^N-enrichment of the diatoms cells during incubations was 2.37 and 71.17 atom%, respectively (measured during nanoSIMS analyses, see below). During the transport of cultures from the laboratory to the sampling station of Western Africa, cultures were mostly kept in darkness at ambient temperature (~ 10–20 °C).

Seawater containing environmental microbial community was sampled with a CTD-Rosette Sampler (CTD: Sea-Bird SBE 9) at 50 m water depth on January 24th, 2012 aboard the RV Poseidon (POS 425) off Cape Blanc, Mauretania (N 20°40,04′ W 18°20,00′, max. water depth 1355 m). Water temperature was 18.3 °C, salinity 36.2, and chlorophyll *a* concentration of approximately 0.6 μg L^−1^. The sampling depth was within the mixed surface layer at the base of the euphotic zone, with the thermocline being located at 90 m. Once environmental microbes were collected, aliquots of the diatom cultures were introduced in 10 L of non-filtered in situ sampled seawater, containing environmental microbial cells. The diatom suspension was filled into four replicate roller tanks with 1.15 L each (cylinder, diameter = 14, height = 7.5 cm), to induce the aggregation of the isotopically-labelled diatoms, and their colonization with natural bacterial populations. Tanks rotated (2 rpm) at in situ temperature and in darkness.

Aggregates were sampled over three days from each tank and at different time points (after 0, 3, 6, 9, 15, 21, 30, 39, 48, 60, and 72 h) by picking individual aggregates using a Pasteur pipette and placing them onto 0.2 µm polycarbonate filters. Between 20 and 30 aggregates were picked for each time-point. The aggregates collected onto polycarbonate filters were resuspended in 0.2 µm-filtered seawater, preserved with paraformaldehyde (PFA, final conc. 1.5%), and stored in the fridge (4 °C) for 24 h. Thereafter, samples were filtered onto polycarbonate membrane filters (0.22 μm GTTP, 25 mm, Merck Millipore). Cells were washed with 96% Ethanol, air-dried, and frozen at -20 °C until CARD-FISH and nanoSIMS analyses.

### Catalyzed reporter deposition-fluorescence in situ hybridization (CARD-FISH)

CARD-FISH analysis was performed following the protocol from Pernthaler et al.^64^. To analyze microbial abundance, a small piece of the polycarbonate filters was cut. This filter piece contained several intact aggregates and their associated bacteria, which were used to determine the abundance of different microbial groups by CARD-FISH. The filter cuts were dipped in 0.1% low melting-point agarose to avoid detachment of the cells from the filter surface. Cell permeabilization was performed by incubation of the filters in lysosome (Fluka, Taufkirchen, Germany) solution (10 mg mL^−1^ lysosome, 0.05 M EDTA [pH 8.0], and 0.1 M TrisHCl [pH 7.5]) for 1 h at 37 °C and in achromopeptidase (60 U mL^−1^, 0.01 M NaCl, 0.01 M Tris–HCl, pH 7.6, Sigma-Aldrich), for 30 min at 37 °C. Hybridizations were carried out in a rotating incubator at 35 °C for 12 h using Horseradish Peroxidase (HRP) labelled probes (50 ng µL^−1^) diluted in 900 µL of hybridization buffer at different formamide (FA) concentrations. The probes and formamide concentrations used are described below; EUB I, II & III used in a mix for bacteria^65,66^ (35% FA), ALT1413 and PSA184^67^ (50% FA) for *Alteromonas* and *Pseudoalteromonas*, CF319^68^ (55% FA) for *Bacteroidetes* and ALF968^69^ (35% FA) for *Alphaproteobacteria*. Hybridized samples were washed with a pre-warmed washing buffer (1 M Tris–HCl, 0.5 M EDTA, 5 M NaCl, and 20% SDS) for 20 min at 37 °C. Sample amplification was done using tyramides labelled with Alexa 488 dye (1 mg mL^−1^, ThermoFisher, Germany) for 30 min at 46 °C. Cell counting was performed by mounting the filters on slides and counterstained with 1.0 μg/mL DAPI (4′,6′-diamidino-2-phenylindole) solution embedded in Citifluor and Vectashield (4:1 vol/vol). Slides were stored in the dark at − 20 °C and examined using a Zeiss Axio-vision epifluorescence microscope (Carl Zeiss, Jena, Germany). Counting was performed at 1000 × magnification on a field of view of 6000 µm^2^. At least 8 fields of view were analysed for each taxonomic group (*Alteromonas* and *Pseudoalteromonas*) at each time point (11 fields of view on average). Bacterial cells were considered as attached when directly in contact with a diatom or in contact with another bacterium which was in contact with a diatom. CARD-FISH enumerations are usually expressed relative to DAPI stained bacterial cells. In our case, within diatom aggregates, the DNA fluorescence derived from DAPI staining of the large nuclei of diatom cells hindered the fluorescence of bacteria preventing exhaustive enumeration. Therefore, the total bacteria abundances were estimated relative to the hybridized cells using the mix of EUB I, II & III probes which has been demonstrated to detect > 99% of prokaryotic cells in the sampled region^66,70^. Microscopic investigation was performed using a Zeiss AxioImager.Z2 epifluorescence microscope equipped with an HXP R 120W/45C UV Hg-vapor lamp, Colibri.2 LED illuminations and the following fluorescence filters: DAPI (365/10 nm excitation, 420 LP emission, FT 395 Beam Splitter), Alexa488 (472/30 excitation, 520/35 emission, 495 Beam Splitter) and Alexa594 (562/40 excitation, 624/40 emission, 593 Beam Splitter). The filter sets used, discriminated clearly between DAPI (365 nm), rRNA hybridization by CARD-FISH (Alexa 488) and natural autofluorescence of the diatom cells. Imaging was done with 100X oil objective numerical aperture N:A 1.4. The software used for image acquisition allows for overlapping images acquired successively with the three different filter sets (Zen software from Carl Zeiss).

In addition to epifluorescence microscopy, Helium Ion Microscopy (HIM, ZEISS) images were also taken. Area of interest containing attached bacterial cells were selected for HIM imaging prior to the nanoSIMS analysis. Analytical conditions employed during HIM imaging are depicted on the individual images. Sample preparation prior to HIM image involves chemical fixation by paraformaldehyde 1.5% at 4 °C for 24 h followed by repetitive washing step in PBS 1X followed by short dehydrations in 96% followed by air drying and storage at − 20 °C until imaging. Parallel filter pieces (metal coated 0.2 µm polycarbonate filters GTTP type, Merck, Millipore) containing diatom aggregates collected for the nanoSIMS analysis at various time points were selected for HIM imaging.

### NanoSIMS analysis

For the nanoSIMS analysis, additional cuts from the same polycarbonate filters used for the previous CARD-FISH analysis were collected. Prior the nanoSIMS, an additional CARD-FISH analysis was performed in order to locate the *Alteromonas* and *Pseudoalteromonas* cells that we were going to analyze with the nanoSIMS. Filters containing ^15^N and ^13^C labelled phytoplankton cells and bacteria were hybridized using the HRP-labeled ALT1413 and PSA189 oligonucleotide probes as described above. The amplification step was done using fluorine containing fluorescently labelled tyramides with Oregon Green® 488 (ThermoFisher, Germany) according to Musat et al.^71^. Diatom cells and hybridized bacteria were mapped using a laser microdissection system (LMD, Zeiss, Germany) equipped with an inverted epifluorescence microscope. The LMD marked areas were further analyzed correlatively using a nanoSIMS 50 L (Cameca, Gennevilliers, France). A 2 pA primary Cs^+^ ion beam of 16 keV was focused into about 70 nm spot at the sample surface during the analysis. Samples were analyzed in a 512 × 512 pixel raster over 30 × 30 µm^2^ areas with 5 ms dwell time per pixel. Before the analysis, the sample surface was treated with 10 nA of low-energy (50 eV) Cs^+^ beam over 80 × 80 µm^2^ area for 10 min. The secondary ions species were analyzed for their mass to charge ratio (m/z) and detected using the seven available collectors: ^12^C^−^, ^13^C^−^, ^19^F^−^, ^12^C^14^N^−^, ^12^C^15^N^−^ and ^13^C^14^N^−^, ^31^P^−^, ^32^S^−^. Two secondary ion species (^12^C^15^N^−^ and ^13^C^14^N^−^) were detected by switching the deflector voltage in combined analysis mode. The mass resolving power (MRP) was checked to be between 7000 and 10,000 with the exit slit width of 70 µm, entrance slit width of 20 µm, aperture slit of 200 µm and with the energy slit cutting about 30% of secondary ions in high-energy tail of their energy distribution. Under these analysis conditions, we observed that cells were sputtered completely within 10 scans. Scans 3–8 were considered for the analysis employing LANS software^72^ allowing for accumulation of the scans with lateral drift correction and quantitative analysis of isotopic ratios (^13^C/^12^C, ^13^C^14^N/^12^C^14^N and ^12^C^15^N/^12^C^14^N). To determine cell-specific isotope ratios, regions of interest (ROIs) were drawn inside the cells, avoiding the cell-surrounding areas.

### Data presentation and statistical analyses

The C and N cellular isotope composition measured by nanoSIMS is presented as fractional isotope abundances (atom%):

1 & 2 $${A}_{13C}= \frac{{}^{13}C}{{}^{12}C+{}^{13}C}*100 \, {\mathrm{or}} \, {A}_{15N}= \frac{{}^{15}N}{{}^{14}N+{}^{15}N}*100$$

The addition of non-isotopically enriched C and N molecules as well as potential leakage of labelled small-molecular-weight cellular components during various chemical steps of the CARD-FISH treatment leads to an isotopic dilution of the analyzed cells^73,74^. In order to correct for this effect, diatoms and bacterial cells were analyzed by nanoSIMS with and without CARD-FISH treatment. ^13^C dilution factors were 2.5% and 29.7% and ^15^N dilution factors were 6.7% and 53.4% for diatoms and bacteria respectively. All the fractional isotopes abundances presented here are corrected from CARD-FISH isotopic dilutions.

At each time point, the proportion of DDC and DDN in bacteria was calculated as follows:

3 $$DDC \, or \, DDN=\frac{{A}_{cell}-{A}_{t0}}{{A}_{diatoms,mean}-{A}_{t0}}$$

With ${A}_{cell}$ being the fractional isotopic abundance of bacteria cells at each time point and ${A}_{t0}$ the mean fractional isotopic abundance in non-labelled samples. The isotopic dilution of diatoms throughout the experiment (see “Results” section) was accounted for by using the average fractional isotopic abundance.

## Supplementary Information

Supplementary Information.

## Data Availability

The authors declare that all data supporting the findings of this study are available within the article and its Supplementary Information file, or from the corresponding author upon request.
