# Supplementary material for: Efficient carbon and nitrogen transfer from marine diatom aggregates to colonizing bacterial groups
Source: Sci Rep. 2022 Sep 2;12:14949. doi: 10.1038/s41598-022-18915-0 (PMC9440002; doi:10.1038/s41598-022-18915-0)
Supplement: Supplementary file 1 — Supplementary Information. [file 41598_2022_18915_MOESM1_ESM.docx]

**Supplementary material**

**Supplementary table 1.** Average abundance of different bacterial groups at each incubation time-point. Abundances were calculated for both, attached and non-attached fractions.

| **Time (hours)** | **0** | **3** | **6** | **9** | **15** | **21** | **30** | **39** | **48** | **72** |
| --- | --- | --- | --- | --- | --- | --- | --- | --- | --- | --- |
| Abundance non-attached fraction (cell mL^-1^) | | | | | | | | | | |
| Alteromonas | 0 | 0 | 0 | 0 | 1687 | 3323 | 4780 | 16307 | 15463 | 7810 |
| Pseudoalteromonas | 0 | 1278 | 281 | 1125 | 7591 | 9059 | 34019 | 128485 | 14901 | 19056 |
| Bacteroidetes | 879 | 433 | 0 | 1125 | 281 | 15182 | 1125 | 843 | 0 | 625 |
| Alphaproteobacteria | 2530 | 230 | 1125 | 3093 | 7872 | 43297 | 562 | 562 | 1968 | 5061 |
| Abundance attached fraction (cell mL^-1^) | | | | | | | | | | |
| Alteromonas | 0 | 0 | 0 | 843 | 562 | 4089 | 6185 | 54543 | 17806 | 18118 |
| Pseudoalteromonas | 0 | 256 | 1687 | 3374 | 20805 | 23741 | 104587 | 35987 | 42454 | 56542 |
| Bacteroidetes | 176 | 12 | 0 | 6748 | 281 | 562 | 0 | 1687 | 1968 | 1250 |
| Alphaproteobacteria | 843 | 77 | 562 | 1968 | 3936 | 5623 | 5342 | 562 | 7310 | 5623 |

**Supplementary table 2.** Average values of Diatom derived Nitrogen (DDN) and Carbon (DDC) for free-living and attached fractions of *Alteromonas* and *Pseudoalteromonas* at each time point.

| Time | 21h | 30h | 48h | 60h | 72h |
| --- | --- | --- | --- | --- | --- |
| DDN non-attached (%) | | | | | |
| Alteromonas | 76.3±10.5 | 95.7±8.3 | 43.7±4.5 | 30.6±2.8 | 63.3±11.6 |
| Pseudoalteromonas | 34.7±18.5 | 43.1±7.9 | 31.1±17.6 | 37.2±16.3 | 44.8±5.1 |
| DDC non-attached (%) | | | | | |
| Alteromonas | 3.2±0.3 | 9.9±1.2 | 6.1±0.7 | 0.5±0.2 | 8.1±1.1 |
| Pseudoalteromonas | 13.3±2.4 | 20.5±2.7 | 14.7±2.5 | 10.3±1.5 | 19.1±3.5 |
| DDN Attached (%) | | | | | |
| Alteromonas | 121.7±9.9 | 108.5±13.6 | 90.2±28.1 | 66.5±10.4 | 77.8±10 |
| Pseudoalteromonas | NA | 37.7±11.4 | 71.0 | 46.8±20.6 | 78.8±18.2 |
| DDC Attached (%) | | | | | |
| Alteromonas | 32.9±4.6 | 20.4±3.4 | 30.4±6.2 | 24.4±3.3 | 15.1±2.3 |
| Pseudoalteromonas | NA | 19.1±2.8 | NA | 22.7±3.7 | 34.1±5.8 |

**Supplementary Figures**


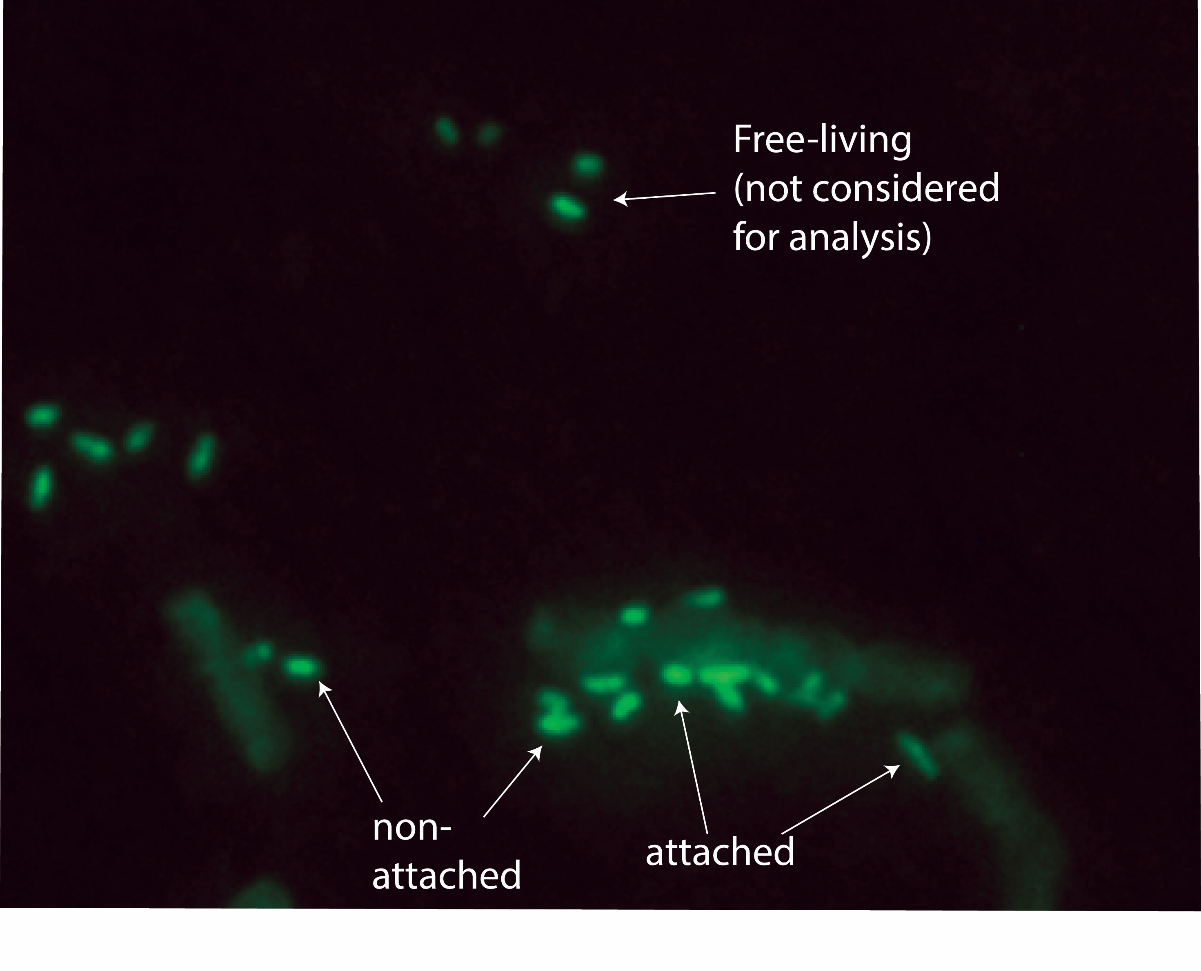


**Supplementary figure 1.** Epifluorescence image displaying the different lifestyles detected: 1- non-attached cells, indicating the bacteria that are in close proximity of the aggregates but with no physical attachment. 2- attached bacteria, showing cells that are within the aggregate and physically attach to the aggregate. 3- free-living bacteria, cells found in the empty space between aggregates. The free-living cells were not considered in this study.


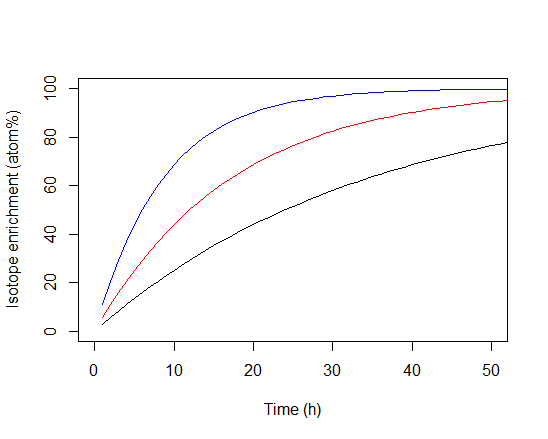
**Supplementary figure 2.** Modelling the isotopic enrichment of cells growing on a unique isotopically labelled source as a function of time at growth rates of 1 d^-1^ (black line), 2 d^-1^ (red line) and 4 d^-1^ (blue line). At growth rates of >4 d^-1^ >95% of the cells reach the isotopic equilibrium with the source already after 21 h of incubation.


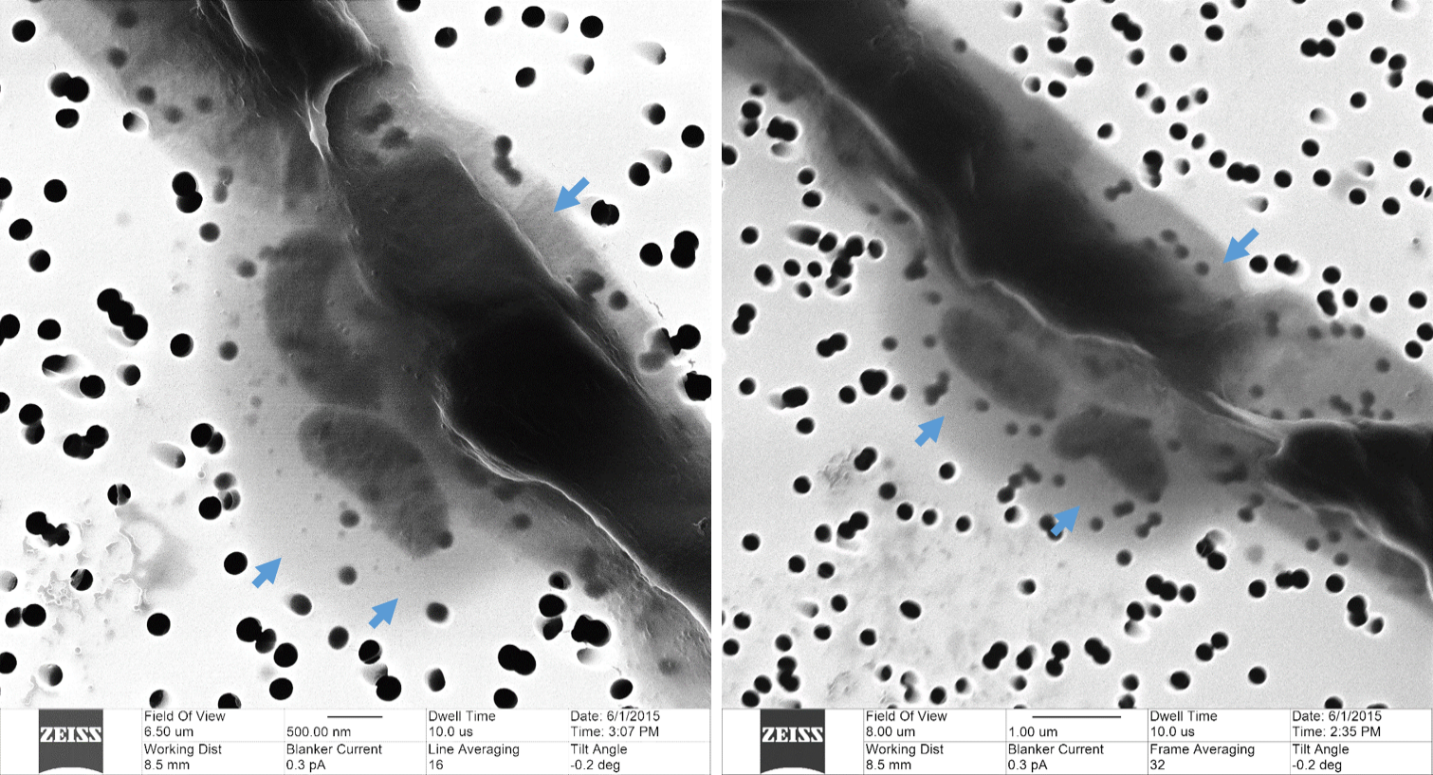


**Supplementary figure 3.** Helium Ion Microscopy image showing diatoms and associated bacteria within an aggregate at time point 72h. Physically attached bacteria can be observed embedded in a translucent matrix (blue arrows), which may indicate transparent exopolymer particles produced by phytoplankton are responsible of aggregate formation while retaining non-attached bacteria within the aggregates. HIM images, courtesy of Matthias Schmidt, UFZ Leipzig.
